# An Audit of An Intensive Care Unit of A Tertiary Care Hospital

**DOI:** 10.31729/jnma.3703

**Published:** 2018-08-31

**Authors:** Subhash Prasad Acharya, Adheesh Bhattarai, Binita Bhattarai

**Affiliations:** 1 Department of Anesthesiology, Institute of Medicine, Tribhuvan University, Maharajgunj, Kathmandu, Nepal

**Keywords:** *audit*, *intensive care unit*, *mortality*, *outcome*, *tertiary level*

## Abstract

**Introduction:**

The patients with the most severe and life threatening illnesses requiring constant monitoring and support are admitted in the intensive care unit. Tribhuvan University Teaching Hospital is the oldest and tertiary referral center hospital in the country with eleven-bedded level III mixed medical surgical ICU. This audit was performed to study the profile of critically ill patients under different headings like diagnosis at admission, primary specialty, outcome of the patients, etc.

**Methods:**

A descriptive cross-sectional study was designed and all patients admitted to the intensive care unit of our center between 13 April 2017 and 13 April 2018 (1^st^ Baisakh 2074 to 30^th^ Chaitra 2074) were selected for this study.

**Results:**

A total of 813 patients were admitted in TUTH ICU over a period of one year (2074 B.S.) with male patients being more common. Neurosurgical cases were most common at 199 (24.48 %), followed by respiratory cases at 149 (18.32%) and so on. The overall mortality was 246 (32.8%). The overall surgical cases were more common than medical cases with better outcomes.

**Conclusions:**

This audit presents the profile of patients admitted in a tertiary level hospital in Nepal, their indications and mortality. The most common patients being admitted were Neurosurgical patients and the mortality was significantly higher compared to ICUs in developed countries.

## INTRODUCTION

An intensive care unit (ICU) is a specialized unit where patients with severe and life-threatening illnesses which require constant monitoring and support are managed. A group of highly skilled doctors and nurses, along with sophisticated equipment are required to properly manage patients in the ICU. Patients from all departments are admitted in the intensive care unit, which requires a broad range of knowledge. The ICU at Tribhuvan University Teaching Hospital (TUTH) is a semi-closed, eleven bedded mixed medical-surgical unit.

Clinical audit plays a vital role in clinical governance and also forms the stepping-stone for quality improvement projects at the heart of which is patient care. Traditionally, audit focused mainly on measuring performance against set standards before making a change in practice and repeating the whole process or closing the loop.^1^ This study is being carried out to retrospectively study the various patients in Tribhuvan University Teaching Hospital ICU (TUTH ICU) for a period of one year.

## METHODS

After ethical approval was taken from Institutional Review Committee at the Research Department of Institute of Medicine, Tribhuvan University, a

descriptive cross sectional study was designed and all patients admitted in the intensive care unit from 1^st^ Baisakh 2074–30^th^ Chaitra 2074 (13 April 2017 till 13 April 2018) over a period of one year were included in the study. The data was obtained by review of ICU Admission Discharge book and electronic medical record and patient charts.

The variables studied were age, sex, diagnosis, preoperative or postoperative, length of ICU, length of mechanical ventilation, outcome (survivor, non-survivor, LAMA), etc. The data were recorded and statistical analysis was done using SPSS Software for windows.

## RESULTS

**Figure 1.**
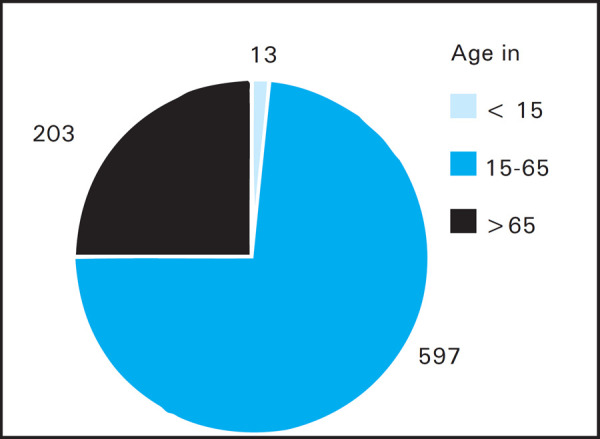
Age distribution of patients (percentage).

During the period of the study a total of 813 patients were admitted in the intensive care unit of which most were admitted from the operation theatre or emergency. Among them, 421 (51.78%) patients were male and 392 (48.22 %) patients were female. Age wise distribution of patients is shown (Figure 1).

**Figure 2.**
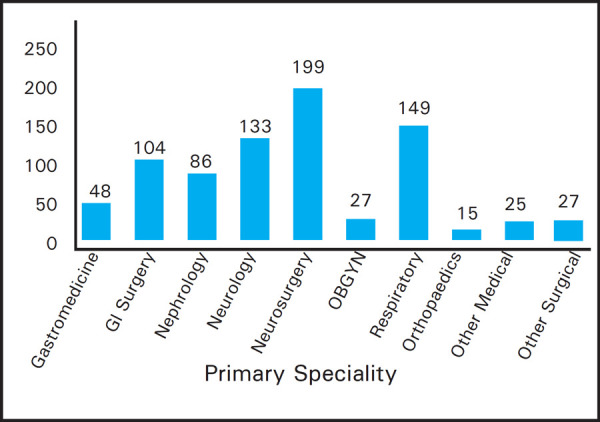
Distribution of patients admitted to ICU based on primary specialty.

Out of these, there were a total of 441 (54.24%) medical patients and 372 (45.76%) surgical patients. Majority of the patients admitted in TUTH ICU were neurosurgical cases, followed by respiratory, neurology, nephrology and general surgery. Distribution of patients based on primary specialty is mentioned (Figure 2). Amongst the Neurosurgical patients, out of 199, 170 (85.42%) of them were postoperative patients.

**Figure 3.**
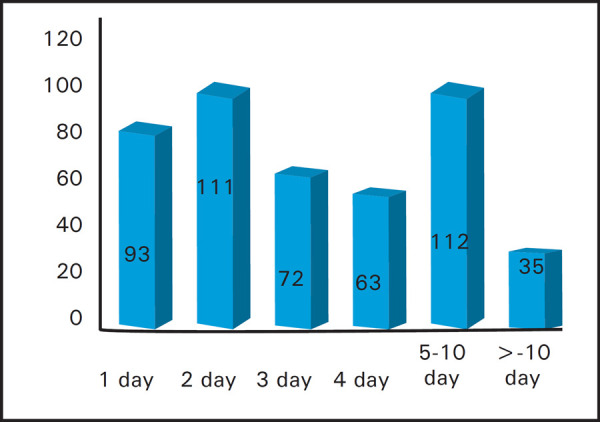
Average Length of Stay on Mechanical Ventilation (LOS MV).

Out of all patients, there were a total 469 patients who were intubated and required mechanical ventilation (57.68%). The average length of stay in ICU (LOS ICU) was 4.5 days whereas the average length of stay in Mechanical Ventilator (LOS MV) was 5.4 days. The average length of patient on Mechanical Ventilation is shown (Figure 3).

**Figure 4.**
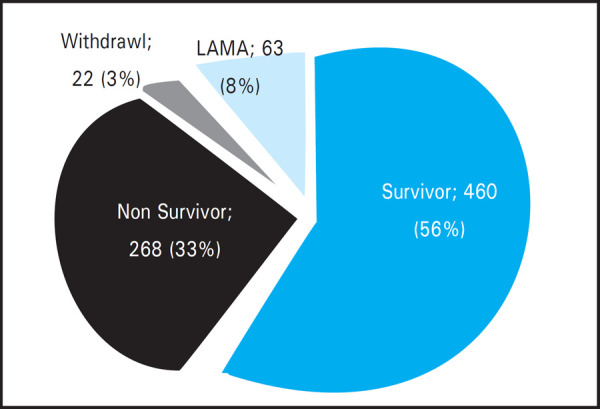
Outcome of patients in TUTH ICU.

Considering the outcome of these 813 patients in TUTH ICU, 460 (56.58%) improved and survived till ICU discharge, 268 (32.96%) expired in ICU among which 22 (2.70%) expired after withdrawal of life support and 63 (7.74%) patients left the hospital against medical advice (LAMA) (Figure 4). Thus the overall mortality in ICU was 246 (32.8%).

Out of 460 patients survived, most of them 382 (83.04%) were transferred out to the post-operative ward, which is a level II step down unit and is being managed by Critical Care Outreach Team. The remaining patients were discharged out of ICU to other step down units or to the floor under the care of primary admitting team.

Amongst non-survivors, 168 (38.09%) patients were with medical disease and 107 (28.76%) patients were with surgical conditions. This higher mortality rates could be because of the sicker nature of patients that are received in TUTH ICU, as this is the tertiary referral center where all complicated patients are referred.

Also, mortality was only 3 (23.07%) in those aged below 15 (out of 13), 181 (30.31%) in those aged 15 to 65 years (out of 597), and 84 (41.37%) in those aged more than 65 years (out of 203).

Outcome of the patients according to their primary specialty is shown (Table 1).

**Table 1:** Outcome of patients according to primary specialty.

	Improved	Mortality	LAMA	Withdrawal	Total
Gastromedicine	19	24	4	1	48
Nephromedicine	39	43	4	0	86
Neurology	75	36	15	7	133
Respiratory	78	52	17	2	149
Other Medical	14	9	1	0	25
Neurosurgery	132	43	15	9	199
Gl Surgery	53	44	6	1	104
Obstetrics and Gynaecology	19	7	0	1	27
Orthopedics	12	3	0	0	15
Other Surgical	19	7	1	1	27
Total	454	275	63	22	813

The best outcome was seen with neurosurgical cases while patients primarily admitted for renal problems had the worst outcomes with mortality of around 43 (51.16%).

## DISCUSSION

Patients admitted in the ICU require multi-disciplinary approach to management, which may require extensive tests and medications. In countries with limited resources, financial constraints also significantly determine the patient outcome. TUTH ICU being one of the tertiary referral centers in Nepal, it is well equipped to manage most of the patients but despite this and our best efforts, the outcome of critically ill patients still remains poor in comparison to developed countries.

This audit presents the profile of patients admitted in a tertiary Level III ICU of Nepal, TUTH ICU, which shows that the admissions are predominantly medical (54.4%) whereas amongst the surgical patients, Neurosurgical patients were the most commonly admitted amounting to 53.49% of all surgical patients and 24.47% of all admission. This was a bit different to other ICU in Nepal were admission were mostly medical (72.4%).^3^ The admission pattern and indications is a bit different to audit from Nigeria^4^ and other parts of Africa, which has practically been described as surgical ICUs^5^ and UK where 60–70% of ICU admissions were surgical.^6^

Among these 813 patients, 421 (51.78%) patients were male and 392 (48.22%) patients were female, which was almost similar to another ICU in a study by Vaidya PR et al.^3^ There were 268 (36.81%) deaths in the ICU, which is higher than in other developing countries^4,5^ and other ICU in Nepal as published by Vaidya PR et al.^3^ The overall survival of patients in this TUTH ICU decreased with increase in age and length of ICU stay. Mortality was highest in those aged more than 65 with 41.37%. The outcome of our patients with mortality of around 33.82% seems a bit high in comparison to ICUs in developed countries but in regard to developing countries, it seems to be similar to other developing countries.^7,8,9^ This may be due to critical condition of patients admitted to our center where very sick patients are referred from all across the country.

Survival of patients mainly depended upon the severity of their illness at ICU admission and presence of any comorbidity. The high mortality can be attributed to the severity of illness on admission and shows a need for scoring the severity of illness at admission. Also, the high incidence of hospital-acquired infections could have contributed to the higher mortality but due to lack of electronic medical system and proper data recording facility, the incidences could not be documented. In TUTH ICU, the best outcome was found in patients following elective surgeries amongst which also neurosurgical patients had the best outcomes. The outcomes of the surgical patients were better than the medical patients and our are similar to the study done by Vaidya PR et al ^3^ and MHM Delwar H et al.^10^ Patients admitted primarily for renal problems had the worst outcome followed by patients with respiratory illnesses.

Surprisingly, 63 (7.74%) patients were taken from the hospital against medical advice (LAMA), which is a significant number amongst which 41 (65.07%) patients were with medical disease and 22 (34.92%) patients were with surgical conditions. Most of these were due to the financial burden on the patient's family.^7,11^ Though TU Teaching Hospital has provision for free beds to treat poor patients, the facility provided in these free beds (that includes bed charge, service charges and investigation charges) are free to the patient but doesn't provide medications. Thus, inability to afford medications also led to some patients being taken from hospital earlier than expected or not being admitted to ICU at all. This study was also similar to audit published by Vaidya et al from Bir Hospital Kathmandu and Koirala et al from BPKIHS at Dharan in 2011.^3,7^

Considering the discharge of patient out of ICU, the presence of Postoperative ward as a step down unit, which is also managed by Critical Care Outreach Team of the ICU, has also facilitated admission and discharge of patients from level III ICU. Whenever a Level III ICU bed was not present, the patient was managed in Level II Postoperative Ward (Step Down Unit). Such provision of Critical Care Outreach Team managing step down units and its role in reducing mortality and facilitating ICU admission and discharge has already been shown by various evidences.^12^

One of the main limitation of the study is that as this data is based on data entry done on a regular basis in the ICU, there might have been some errors if the data has been entered in a wrongly manner. This problem can be solved by the use of electronic medical records with appropriate softwares and thus ICUs and hospitals should also plan to move to digitalizing medical records.

## CONCLUSIONS

General medical patients remains the main bulk of ICU patients in tertiary level ICU of TU Teaching Hospital and Neurosurgical patients are the commonest surgical patients requiring ICU with lowest mortality amongst all ICU admissions. The mortality of this ICU seems a bit higher given the complex nature of patients the hospital receives being the tertiary referral center from all over the country.
